# Effect of Vegetable Juices on Properties of Two Resin Composites Used for Dental Caries Management

**DOI:** 10.3390/medicina59040774

**Published:** 2023-04-16

**Authors:** Claudia Tighiceanu, Elena Raluca Bulai, Oana Camelia Iatcu, Constantin Dulucheanu, Alexandru Nemtoi

**Affiliations:** 1 Integrated Center for Research, Development and Innovation in Advanced Materials, Nanotechnologies, and Distributed Systems for Fabrication and Control, Stefan cel Mare University of Suceava, 13 University Street, 720229 Suceava, Romania; 2 Faculty of Medicine and Biological Sciences, Stefan cel Mare University of Suceava, 13 University Street, 720229 Suceava, Romania; 3 Faculty of Mechanical Engineering, Automotive and Robotics, Stefan cel Mare University of Suceava, 13 University Street, 720229 Suceava, Romania

**Keywords:** resin composites, colour change, vegetable juices, caries management, microhardness

## Abstract

*Background and Objectives*: Resin composites represent a class of materials widely used in restorative dentistry due to patient demands for better aesthetics. Colour changes in composite resins can occur due to intrinsic and extrinsic factors. Beverages, such as vegetable juices, can be some of these extrinsic factors. The purpose of this study was to investigate the colour stability and modification of microhardness of two resin composites before and after immersion in different vegetable juices. *Materials and Methods*: The colour of two resin composite materials (Gradia Direct Anterior-shade A2 and Valux Plus—shade A2) was measured before and after immersion in four different solutions: distilled water (as control), beetroot, carrot, and tomato juice. Colour values (L *, a *, b *) were measured over a white background with a colorimeter, using the CIE L *a *b * system. Colour change values were calculated after 1, 3, 5, and 7 days of immersion. Microhardness measurements were taken before and after seven days immersion in test media. Repeated measures analysis of variance (ANOVA) and independent *t*-tests were applied for statistical analysis. *Results:* All vegetable juices produced statistically different discolouration after immersion for 7 days (*p* < 0.05). Tomato juice produced the most discolouration for the Gradia Direct specimens, whereas beetroot juice and carrot juice produced the most discolouration for the Valux Plus specimens. Microhardness of the materials immersed for 7 days in vegetable juices was reduced compared to the ones immersed in distilled water. *Conclusions:* Vegetable juices, immersion time, and dental resin composites are significant factors that may affect colour stability and microhardness of composite resins.

## 1. Introduction

Dental restorative materials are used to replace a decayed portion of tooth structure due to dental cavities, dental traumas, or tooth wear, and UV light is commonly used by dentists to cure fillings [1]. Among these materials, the use of composite resins has increased recently due to increasing demands for aesthetic restorations. Besides the aesthetic appearance, physical and mechanical properties of these materials are also of great importance. Any aesthetic restorative material must simulate tooth surface texture and its natural colour and also maintain these characteristics for long periods of time. However, discolouration of dental materials remains a major problem for their long-term use [2,3,4,5,6,7].

Discolouration of restorations can be caused by two factors: extrinsic and intrinsic. Intrinsic staining is permanent and can be related to the matrix, type, and amount of filler; photoinitiator system; and polymerization degree (conversion rate) of the composite resins. Extrinsic factors include surface stains, accumulation of plaque, superficial degradation, or a slight penetration of staining agents in the superficial layers of dental materials which can lead to alterations of surface or subsurface colour. Moreover, externally induced discolouration can be associated with surface integrity and surface roughness, which is related to the finishing and polishing technique [4,5,6,7,8,9,10].

Dental materials are inevitably exposed to food and drinks which may cause a colour change and may affect the aesthetic and physical properties. The degree of discolouration is affected by oral hygiene, smoking, and eating–drinking habits. Previous studies on colour stability and surface properties of composites have shown that different beverages, such as coffee, tea, red wine, juices, and carbonated drinks, may affect not only the aesthetic but also the physical properties of composite resins, thereby undermining the quality of the restoration. The effect of beverages on the properties of composite resins may also be directly related to the amount and frequency of their intake [4,8,9,11,12,13,14,15,16]. Several studies suggest that nanohybrid composites do not exhibit superior stain resistance compared to microhybrid composites when immersed in different beverages. Therefore, the chosen materials for this study were a microhybrid and a hybrid composite [15,16,17]. Consumption of 100% fruit juice beverages is an easy way to increase the nutrients in the diets of adults and children. Besides fruit juices, vegetable juices are also commonly consumed. Juices (even vegetable juices) contain naturally occurring acids that can lead to tooth decay, dental erosion, or colour changes. Among vegetable juices, beetroot, carrot, and tomato juices were selected as they are frequently introduced into adults’ and children’s diets; are known to be high in antioxidants, easily accessible, and affordable; and have a strong potential to stain tooth restorative materials [18].

The aim of this study was to evaluate the effects of immersion in different vegetable juices (beetroot, carrot, and tomato juices) on the colour and microhardness of two resin-based composite materials. The null hypotheses tested were: (i) vegetable juices have no effect on the colour stability of the resin composites chosen for the study at different times, and (ii) there are no differences in microhardness between resin composites immersed in vegetable juices for 7 days and the ones immersed in distilled water.

## 2. Materials and Methods

### 2.1. Materials and Sample Preparation

Two resin composite materials were used in this study: micro-filled hybrid resin composite Gradia Direct Anterior, shade A2 (GC Corporation, Tokyo, Japan, Lot number 2102191) and hybrid resin composite Valux Plus, shade A2 (3M ESPE, USA, Lot number NE88327). The light source used to light cure the specimens was Dental Curing Light (Curing Pen, Eighteeth, Changzhou Sifary Medical Technology Co., Ltd., Changzhou, China). The storage environments were commercially available 100% vegetable juices (beetroot juice, carrot juice, and tomato juice) (Josef Polz, Garching, Germany), and distilled water was used as the control.

A total of 72 samples (36 samples for each material), 14 ± 0.1 mm diameter and 1.5 ± 0.1 mm thick, were prepared using a circular mould held between two glass slides. A 2 kg weight was placed on top for 1 min to facilitate uniformity of the sample. Samples were cured for 20 s on each side (40 s in total) with a light-curing device set at 1500 mW/cm^2^ at a 2 mm distance. After polymerization, the samples were removed from the mould and polished on one side using KENDA C.G.I., Polishers for Composites, Compomers, and Glass-Ionomers Polishers, Coarse. The final thickness of the polished specimens was 1.4 ± 0.1 mm, which was verified by a micrometre (Mitutoyo, Kawasaki, Japan).

After preparation, all samples were stored in distilled water at 37 °C for 24 h, for water absorption and completion of the polymerisation process, in proximity to oral conditions, using a Forced-Air Drying Oven (BOV V136F, Biobase, Jinan, China). Specimens of each type of composite were then randomly divided into 4 subgroups (n = 9) [9]: group 1 to be immersed in distilled water (which acted as a control group), group 2 to be immersed in beetroot juice, group 3 to be immersed in carrot juice, and group 4 to be immersed in tomato juice, for a total period of seven days at 37 °C. Baseline measurements were made following 24 h immersion in distilled water.

The pH of each beverage was measured before immersion with a pH meter (SevenCompact S210, Mettler–Toledo GmbH, Greifensee, Switzerland) (pH distilled water 6.58 ± 0.1, beetroot juice 4.34 ± 0.06, carrot juice 4.42 ± 0.02, tomato juice 4.26 ± 0.04). Beverages were used at a temperature of about 4 °C, and they were replaced daily to prevent possible interactions/bacterial adhesion.

### 2.2. Colour Stability Testing

The colour was evaluated on the polished side of the specimens using a colorimeter (Chroma Meter CR400, Konica Minolta, Tokyo, Japan) against a white background using the Commission International de I’Eclairage CIE (L *a *b *) colour system [5,8]. The colour was measured before being immersed in beverages as a baseline and also after immersion for 1, 3, 5, and 7 days, respectively. At each time point, specimens were rinsed with distilled water for 20 s after being removed from the solution and blotted dry with absorbent paper. The overall colour change ΔE * was calculated from the single colour values L *, a *, and b *, according to Equation (1) [4,9,12]:

ΔE * = [(ΔL *)^2^ + (Δa *)^2^ + (Δb *)^2^]^1/2^ (1)

ΔL * describes the differences in brightness, Δa * stands for the changes on the red–green axis, and Δb * depicts the shifting on the yellow–blue axis.

As previously described in the literature, the following scale was used to classify the resulting colour difference values (∆E): a difference below 2.0 units in the CIE colour space was classified as unnoticeable, between 2.1 and 3.5 units as slightly perceptible, between 3.6 and 5.0 units as clearly perceptible, and 5.1 units or above as pronounced [11].

### 2.3. Microhardness Measurement

Vickers microhardness values were measured by testing four samples from each group [19] before and after 7 days of immersion in the solutions, using a MicroHardness Tester (DuraScan 70, Emco Prüfmaschinen-Test GmbH, Kuchl, Austria). For every sample, three indentations were made, each being no closer than 0.5 mm to the adjacent indentation (EN ISO 6507-1:2018), and the test load of the Vickers indenter was 4.9 N (0.5 kgf). The two diagonal lengths of each indentation were measured by a 40× magnification built-in scale microscope and were converted into a microhardness value (HV) using the following equation: HV = 1.854 P/d^2^, where HV is microhardness in kgf/mm^2^, P is the load in kgf, and d is the average length of the diagonals in mm.

### 2.4. Statistical Analysis

Data were analysed using SPSS software version 20. Descriptive statistics, including mean, standard deviation of mean, and minimum and maximum values, were calculated for each group. An independent *t*-test was used to compare the data after immersion in vegetable juices and water. To evaluate the differences between the discolouration observed at different immersion time points, repeated measures ANOVA was used. In the present study, *p* ≤ 0.05 was considered as the level of significance. An independent *t*-test was used to compare the data on microhardness of the composite materials following 7 days immersion in vegetable juices and water.

## 3. Results

### 3.1. Effect of Vegetable Juices on Colour Stability of Resin Composites

**Figure 1:**
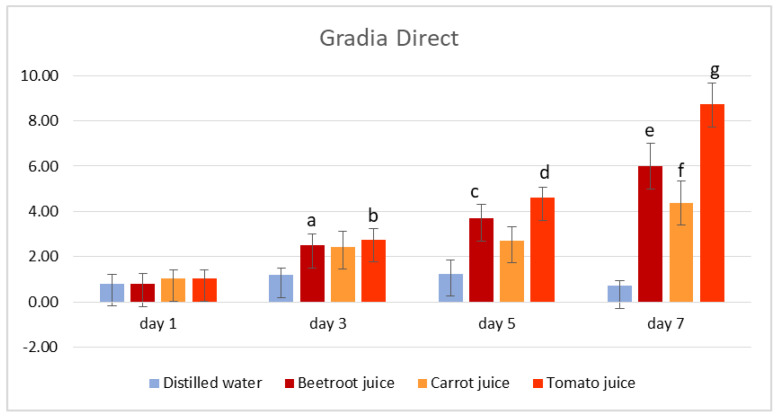
Colour change (mean values ± standard error of mean) for Gradia Direct samples. A lowercase letter indicates a statistical difference between the mean discolouration of samples immersed in distilled water and that of samples immersed in vegetable juice at each time point (e.g., “a” indicates a statistical difference between the mean discolouration of samples immersed in distilled water and that of samples immersed in beetroot juice for 3 days).

**Figure 2:**
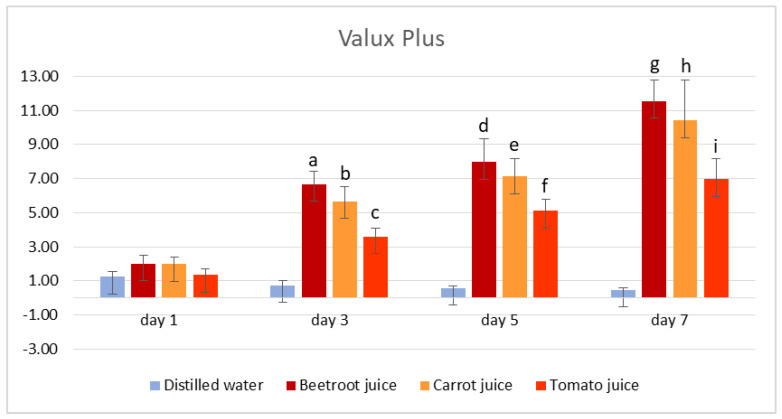
Colour change (mean values ± standard error of mean) for Valux Plus samples. A lowercase letter indicates a statistical difference between the mean discolouration of samples immersed in distilled water and that of samples immersed in vegetable juice at each time point (e.g., “b” indicates a statistical difference between the mean discolouration of samples immersed in distilled water and that of samples immersed in carrot juice for 3 days).

The results for the colour measurements of the tested samples at different time points of immersion and in the different vegetable juices are shown in Tables S1 and S2, which can be found in the Supplementary Material, in Table 1, and in Figure 1 and Figure 2.

For Gradia Direct samples, imperceptible differences with ΔE < 2.0 were noted after immersion in water at all time points and after immersion in all vegetable juices for 1 day. Slightly perceptible differences with ΔE between 2.1 and 3.5 were recorded for samples immersed in all vegetable juices for 3 days and also for samples immersed in carrot juice for 5 days. Clearly perceptible differences with ΔE between 3.6 and 5.0 were observed for beetroot and tomato juices at day 5. A pronounced colour difference was noted only at day 7 for samples immersed in beetroot juice and tomato juice. The highest value of ΔE was recorded from tomato juice as the staining liquid after 7 days of immersion (ΔE = 8.74 ± 0.92).

The statistical analysis of mean colour change ΔE of Gradia Direct samples immersed in vegetable juices versus control (samples immersed in distilled water) for each time point is presented in Table S1. The results showed a statistical difference between the mean discolouration of samples immersed in distilled water and samples immersed in beetroot and tomato juices for 3 days and 5 days, respectively, and also samples immersed in all vegetable juices for 7 days (*p* < 0.05).

A statistical analysis of mean discolouration of Gradia Direct samples immersed in vegetable juices at consecutive time points of immersion was also conducted using repeated measures ANOVA. In beetroot juice, statistically significant differences were observed between day 3 and day 5 (*p* = 0.012) and between day 5 and day 7 (*p* = 0.037). In carrot juice, statistically significant differences were observed between day 3 and day 5 (*p* = 0.013) and between day 5 and day 7 (*p* = 0.002). In tomato juice, statistically significant differences were observed between day 3 and day 5 (*p* = 0.001) and between day 5 and day 7 (*p* < 0.001).

For the Valux Plus samples, similar to Gradia Direct, imperceptible differences with ΔE < 2.0 were noted after immersion in water at all time points and after immersion in all vegetable juices for 1 day. For samples immersed in beetroot juice for 1 day, the average ΔE was 2.01, a value close to the threshold and therefore considered an imperceptible difference. Starting day 3, the effect of vegetable juice on the colour of the samples was more pronounced for the Valux Plus than for Gradia Direct ones. For the samples immersed in beetroot and carrot juices, ΔE was higher than 5.0, and the samples immersed in tomato juice for 3 days showed a clearly perceptible difference with ΔE = 3.60. A pronounced colour difference was noted after 5 days of immersion and also after 7 days for all Valux Plus samples immersed in vegetable juices, with the highest value of ΔE being recorded from beetroot juice as the staining liquid after 7 days of immersion (ΔE = 11.53 ± 1.27).

The results of the independent *t*-test showed a statistical difference between the mean discolouration of samples immersed in distilled water and samples immersed in all vegetable juices for 3, 5, and 7 days, respectively (*p* < 0.05).

The mean discolouration of Valux Plus samples immersed in vegetable juices was statistically analysed over the 7 days by repeated measures ANOVA, between consecutive time points. In beetroot juice, statistically significant differences were observed between day 1 and day 3 (*p* < 0.001) and between day 5 and day 7 (*p* = 0.031). In carrot juice, statistically significant differences were observed between day 1 and day 3 (*p* < 0.001). In tomato juice, differences in the colour of the material were observed between day 1 and day 3 (*p* < 0.001) and between day 3 and day 5 (*p* = 0.007).

An independent *t*-test was used to compare the mean discolouration of Gradia Direct and Valux Plus samples exposed to the same vegetable juice at each time point to see if these materials were affected differently by the test media (Table 1). As shown in Table 1, no significant difference was found between the mean discolouration of Gradia Direct and Valux Plus samples immersed in tomato juice at any time point and for those immersed in carrot juice for 1 day (*p* > 0.05). At 3-, 5-, and 7-days immersion time in carrot juice, results showed a significant difference between the materials (*p* < 0.05). Beetroot juice appeared to be the only vegetable juice tested that affected the two materials differently, with a significant difference being observed at all time points (*p* < 0.05).

### 3.2. Effect of Vegetable Juices on Microhardness of Resin Composites

Microhardness measurements were taken before and after seven days of immersion in test media and reported as mean ± standard error of mean. Microhardness measurements taken before immersion in vegetable juices were 40 ± 0.16 for Gradia Direct and 129 ± 0.55 for Valux Plus samples. Microhardness results for Gradia Direct and Valux Plus samples after seven days of immersion in vegetable juices are reported in Table 2. Results showed that the microhardness of Valux Plus samples was greater than that of Gradia Direct samples. For both materials, microhardness values reduced slightly in all the groups following exposure to vegetable juices compared to those immersed in water. An independent *t*-test was used to evaluate the effect of beverages on microhardness. For both materials, statistically significant differences in microhardness were observed after seven days of immersion in all three types of vegetable juice when compared to those immersed in water (Table 2).

The percentage variation in microhardness of samples immersed for seven days in vegetable juice compared with those immersed in water was evaluated by Equation (2).

Percentage variation in microhardness = (Microhardness in water-Microhardness in juice)/Microhardness in water × 100 (2)

**Figure 3:**
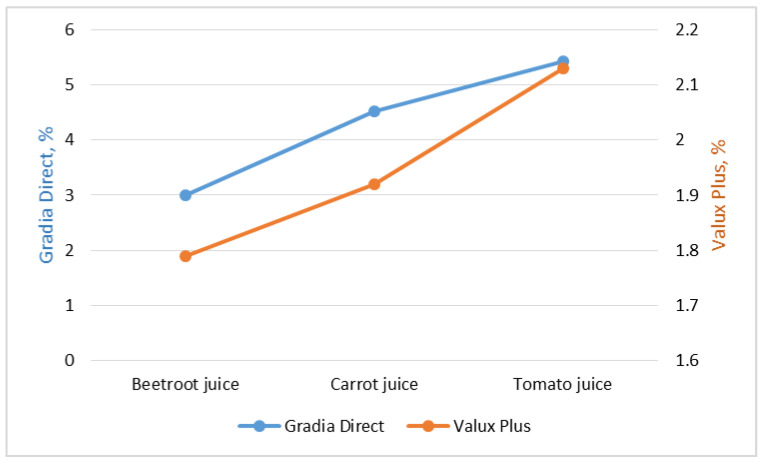
Percentage variation in microhardness for Gradia Direct and Valux Plus samples.

The results obtained are presented in Figure 3. The standard fraction change in microhardness for both materials followed the same pattern, increasing from beetroot juice to carrot juice and tomato juice. Percentage variation in microhardness appeared to be more pronounced for Gradia Direct samples compared with Valux Plus samples, and tomato juice seemed to have a greater effect compared to the other two vegetable juices.

## 4. Discussion

Discolouration of dental restorative materials constitutes a continuous challenge in dentistry and can be a reason for the replacement of dental restorations, especially in aesthetic areas. This process concerns both patients and dentists and is time- and money-consuming.

Colour changes can be evaluated using a visual method, which is subjective and thus of low reproducibility, or by instrumental techniques, which give objective and statistically utilizable results. In dentistry, colour measurement devices are generally utilised, and the Commission International de I’Eclairage CIE (L *a *b *) colour system is used to determine the colour differences or changes.

The results of this study indicated that colour changes varied based on the type of resin composite, time of immersion, and staining liquid. After immersion in vegetable juices, it was observed that the colour differences (ΔE) increased for both resin composites over the experimental period, regardless of staining solution; the most intense colour change occurred after seven days.

Among all the vegetable juices tested, the largest colour change for Gradia Direct samples was observed in tomato juice, followed by beetroot and carrot juices. The largest colour change for Valux Plus samples was seen in beetroot juice, followed by carrot juice and tomato juice. When comparing the two materials, microhybrid Gradia Direct samples generally exhibited the least colour changes, except for samples immersed in tomato juice for seven days where a ΔE value of 8.74 ± 0.92 was recorded compared with ΔE = 6.95 ± 1.22 for Valux Plus. In this study, pH measurements showed similar values for all vegetable juices, and they were in the acidic region.

The colour changes in resin composites after immersion in various liquid beverages has been a subject of great interest in many studies. Factors that influence colour changes can be attributed both to beverages (colourants, pH) and dental materials (composition, properties). Acidic foods and beverages may induce discolouration by affecting surface integrity. The staining susceptibility of resin composites might be related to their degree of water sorption and the hydrophilicity of the matrix resin. The absorption and adsorption of colourants with different polarities, found in different beverages, on the surface of resin composites have also been proposed as potential factors. Additionally, it has been suggested that silanisation of filler particles plays an important role in discolouration due to the fact that silane has high water absorption levels [4,8,9,11,13].

Our findings showed that Valux Plus had generally lower colour stability compared to Gradia Direct. Differences in resin matrix and filler compositions could be factors that determined different discolourations of the two composites. The resin matrix of the Valux Plus product contains Bisphenol A Diglycidyl Ether Dimethacrylate (BISGMA), Triethylene Glycol Dimethacrylate (TEGDMA) and silane treated ceramic, which are considered vulnerable to staining due to their increased hydrophilicity [20,21]. These composites have zirconia/silica fillers [22,23], which may have a porosity that facilitates colourant penetration. These results are in agreement with previous studies that have reported that products that contain similar components in the matrix and fillers, as in Valux Plus, were prone to staining and discolouration [8].

Immersion in different test media can result in the degradation of the matrix and fillers of resin composites, to some extent. Surface hardness can be used as a predictor of the wear resistance of a material [24]. The wear behaviour of the composite resins can be affected not only by the type, size, and distribution of the filler, but also by the matrix and the bonding strength between the matrix and filler. In addition, by increasing the volume fraction of the filler, the wear loss decreases [25,26]. Pre-polymerized fillers have a lower hardness than silica and zirconia mineral fillers [27].

Previous studies have reported that the filler fraction for Gradia Direct is 73% by weight (64% by volume), containing silica and pre-polymerized fillers (average particle size 0.85 μm), and for Valux–Plus, it is 85% by weight (66% by volume), containing zirconia–silica fillers (particle size 0.6–1 μm) [22,23]. In this study, microhardness values for Gradia Direct samples were found to be significantly lower than for Valux Plus samples. This could be associated with the lower amount of filling in Gradia Direct compared with Valux Plus samples, and also with the type of filling, which would be in agreement with previous studies where composites with the highest filler by volume exhibited the highest hardness.

The pH is a very valuable indicator of the body’s health, starting from the oral cavity. Good oral health involves maintaining an environment of the mouth near a neutral pH. An acidic pH in the oral cavity contributes to dental erosion. Previous studies have shown that some acidic foods and beverages can cause surface degradation and reduce surface hardness of restorative dental materials [19,28,29,30,31]. In this study, both tested resin composites registered a slight decrease in microhardness after one week immersion in juices, compared with samples immersed in distilled water (*p* < 0.005). This can be associated with the acidic pH of the beverages, results that are in agreement with previous studies where surface hardness was reduced after immersion in acidic drinks. Considering the results obtained, both null hypotheses were rejected.

The oral cavity is a complex and dynamic environment, and teeth and dental restorations are exposed to a wide range of changes, including thermal or pH changes following consumption of cold, hot, or acidic foods or drinks. Saliva plays also an important role in diluting, neutralizing, and cleaning the oral cavity [32]. Furthermore, beverage and food ingestion are dynamic processes that do not allow for the sustained static retention of fluid in the oral cavity [8]. Therefore, clinical investigations evaluating the real effect of different vegetable juices on colour stability of the aesthetic restorative materials tested in this study may be beneficial. Further investigations may be required to evaluate the effect of different fruit and vegetable juices on colour and mechanical and surface properties of aesthetic restorative materials containing different resin matrices and filler compositions.

In the clinical practice, patients should be aware of the staining effects of the drinks tested in this study, while practitioners should take into consideration the staining susceptibility and the mechanical properties of the resin composites and recommend the ones appropriate to the dietary habits of their patients.

## 5. Conclusions

Within the limitations of this study, the subsequent conclusions were drawn: Immersion in vegetable juices affected the aesthetic and mechanical properties of the resin composites tested. Colour changes were influenced by the composition of the materials, time of immersion, and vegetable juices. For both resin composites, vegetable juices induced discolouration which increased with exposure time. The highest colour change for Gradia Direct samples was observed after immersion in tomato juice, while Valux Plus samples showed the highest discolouration when immersed in beetroot juice. Change in colour was generally higher in Valux Plus samples compared to Gradia Direct samples, except for samples immersed in tomato juice for seven days. The microhardness of the two materials tested significantly differed due to their compositions. Both resin composites immersed in vegetable juices for seven days showed a lower microhardness than those immersed in distilled water.

## Figures and Tables

**Table 1:** *p*-values for independent *t*-test (for comparison of ΔE * for both materials at different time points).

	Day 1	Day 3	Day 5	Day 7
Beetroot juice	0.037 *	<0.001 *	0.011 *	0.004 *
Carrot juice	0.113	0.010 *	0.004 *	0.031 *
Tomato juice	0.540	0.235	0.536	0.260

* Indicates significant difference.

**Table 2:** HV microhardness (Mean ± Standard Error of Mean) of Gradia Direct and Valux Plus samples after seven days of immersion in test media.

	Gradia Direct		Valux Plus	
	Mean ± SEM	*p* Value	Mean ± SEM	*p* Value
Water	39.76 ± 1.20	-	121.33 ± 2.39	-
Beetroot juice	38.57 ± 0.97	0.014 *	119.17 ± 1.27	0.013 *
Carrot juice	37.96 ± 1.44	0.003 *	119.00 ± 1.21	0.008 *
Tomato juice	37.60 ± 0.61	<0.001 *	118.75 ± 1.22	0.004 *

* Indicates significant difference.

## Data Availability

All data are available from the corresponding authors.

## Supplementary Materials

The following supporting information can be downloaded at: https://www.mdpi.com/article/10.3390/medicina59040774/s1, Table S1: Colour change ΔE (Mean ± Standard Error of Mean) for Gradia Direct in different test media at different time points; Table S2: Colour change ΔE (Mean ± Standard Error of Mean) for Valux Plus samples in different test media at different time points.
