# Supplementary material for: Effect of Vegetable Juices on Properties of Two Resin Composites Used for Dental Caries Management
Source: Medicina (Kaunas). 2023 Apr 16;59(4):774. doi: 10.3390/medicina59040774 (PMC10142735; doi:10.3390/medicina59040774)
Supplement: Supplementary file 1 [file medicina-59-00774-s001.zip › medicina-2288292-supplementary.pdf]

Table S1. Colour change ΔE (Mean ± Standard Error of Mean) for Gradia Direct in different test media at different time points

		Day 1	Day 3	Day 5	Day 7
Distilled water	ΔE ± SEM	0.82 ± 0.38	1.19 ± 0.28	1.25 ± 0.59	0.70 ± 0.24
Beetroot juice	ΔE ± SEM	0.80 ± 0.46	2.50 ± 0.51	3.68 ± 0.63	5.99 ± 1.01
	p value	0.975	0.042*	0.013*	0.001*
Carrot juice	ΔE ± SEM	1.03 ± 0.37	2.44 ± 0.68	2.72 ± 0.59	4.38 ± 0.97
	p value	0.698	0.114	0.154	0.005*
Tomato juice	ΔE ± SEM	1.03 ± 0.37	2.76 ± 0.48	4.59 ± 0.47	8.74 ± 0.92
	p value	0.691	0.013*	< 0.001*	< 0.001*

*indicates significant difference

Table S2. Colour change ΔE (Mean ± Standard Error of Mean) for Valux Plus samples in different test media at different time points

		Day 1	Day 3	Day 5	Day 7
Distilled water	ΔE ± SEM	1.24 ± 0.31	0.74 ± 0.26	0.57 ± 0.15	0.48 ± 0.13
Beetroot juice	ΔE ± SEM	2.01 ± 0.47	6.67 ± 0.76	7.98 ± 1.35	11.53 ± 1.27
	p value	0.196	< 0.001*	0.001*	< 0.001*
Carrot juice	ΔE ± SEM	1.99 ± 0.43	5.65 ± 0.85	7.12 ± 1.03	10.40 ± 2.35
	p value	0.182	< 0.001*	< 0.001*	0.003*
Tomato juice	ΔE ± SEM	1.36 ± 0.35	3.60 ± 0.47	5.12 ± 0.68	6.95 ± 1.22
	p value	0.814	< 0.001*	< 0.001*	< 0.001*

*indicates significant difference
